# The effect of therapeutic massage combined with conventional therapy in children with functional dyspepsia: a systematic review and meta-analysis

**DOI:** 10.3389/fphar.2025.1554438

**Published:** 2025-04-16

**Authors:** Shaohong Lin, Ruming Ye, Guanhong Wu, Lixia Wu, Ying Lin, Dan Li, Namei Xie, Huiyue Zhang

**Affiliations:** ^1^ Xiamen Children’s Hospital, Children’s Hospital of Fudan University (Xiamen Branch), Xiamen, China; ^2^ Xiamen Neonatal Quality Control Center, Xiamen, China; ^3^ Jinjiang Municipal Hospital, Fujian, China; ^4^ Fujian Key Laboratory of Neonatal Diseases, Xiamen Children’s Hospital, Xiamen, China

**Keywords:** therapeutic massage, functional dyspepsia, child, randomized control trials, systematic review, meta-analysis

## Abstract

**Background:**

Therapeutic massage has been widely used for functional dyspepsia (FD) in children. Emerging evidence suggests that it serves as an effective complementary therapy for pediatric FD. However, no related systematic reviews have been published to date.

**Objective:**

To conduct a systematic review and meta-analysis to evaluate the effectiveness of therapeutic massage in conjunction with conventional therapy for children with FD.

**Methods:**

A search was conducted across PubMed, the Cochrane Library, and nine additional databases, up to November 2024. We included randomized controlled trials (RCTs) that recruited children with functional dyspepsia. These trials compared therapeutic massage combined with conventional therapy to conventional therapy alone. Dichotomous symptom data were aggregated to calculate the relative risk (RR) of overall response following therapy. Continuous data were aggregated utilizing a standardized mean difference with a 95% confidence interval.

**Results:**

The search identified 1,190 citations. Twelve RCTs were eligible for inclusion, which contained 1,161 patients. The response rate of combination therapy, which includes therapeutic massage alongside conventional therapy, was found to be superior to that of conventional therapy alone. Subgroup analysis indicated that both point massage and chiropractic, when combined with conventional therapy, also demonstrated superior response rates compared to conventional therapy alone. Subgroup analysis of the frequency of intervention over a 2-week duration of therapeutic massage indicated that the overall response rates for combination therapy at frequencies of five times per week and seven times per week were superior to those of conventional therapy. In comparison to conventional therapy, combination therapy markedly reduced symptom scores related to abdominal pain, flatulence, anorexia, eructation, nausea and vomiting, and early satiety. Additionally, it enhanced levels of growth hormone-releasing peptide, neuropeptide Y, motilin, and gastrin, while decreasing levels of 5-hydroxytryptophan.

**Conclusion:**

Results demonstrated that compared to conventional therapy, therapeutic massage combined with conventional therapy can significantly reduce symptoms and enhance gastrointestinal hormone levels in children with functional dyspepsia. However, due to the heterogeneity of the findings and the low quality of evidence, further extensive and methodologically sound trials are necessary to validate whether therapeutic massage can serve as an effective complementary therapy for pediatric functional dyspepsia.

**Systematic Review Registration:**

identifier CRD42024540844.

## 1 Introduction

Functional dyspepsia (FD) is a functional gastrointestinal disorder characterized by recurrent epigastric discomfort and nausea in the absence of significant structural abnormalities in the gastrointestinal tract or other organ systems. FD has become increasingly prevalent in pediatric clinics, affecting approximately five million children in China (3% prevalence) (Smeets et al., 2019; Wei et al., 2021). FD exhibits a prolonged and incurable trajectory, lacking a specific treatment or optimal curative effect at this time, which may impact the growth and development of children. (Doi et al., 2019; Lacy et al., 2019; Pittayanon et al., 2019). Clinical research examining sleep, diet, and stress levels in children with Functional Dyspepsia (FD) compared to healthy children reveals that those with FD experience a higher incidence of sleep disorders, exhibit erratic eating patterns, and report greater mental stress. These findings suggest that FD negatively impacts the growth, development, and overall physical and psychological health of children (Shen et al., 2019; Sun et al., 2018; Wu et al., 2018).

Conventional therapies for FD include cognitive behavioral treatment, dietary modifications, probiotics, Mind-Body Therapy, physical activity interventions, and others (Waseem and Rubin, 2022). Due to the limited therapeutic benefits of conventional therapies, many children with FD are treated with complementary and alternative therapies, such as therapeutic massage, in addition to conventional therapies (Yoon et al., 2022). Therapeutic massage, referred to as Tuina in China, is a method for addressing ailments through the manipulation of specific muscle or soft tissue areas using fingers, palms, elbows, knees, or feet, based on the principles of traditional Chinese medicine (TCM) (Dai et al., 2023). Research indicates the benefits of therapeutic massage in addressing pediatric digestive disorders. Therapeutic massage targets acupoints on the body surface using specific techniques, stimulating meridians to regulate internal organs. This process promotes gastrointestinal peristalsis and the secretion of gastric juice and pepsin, thereby maintaining the normal functioning of the spleen and the stomach’s qi, blood, and transportation functions, which aids digestion and enhances children’s appetites (Ding, 2023).

An increasing number of RCTs have investigated the efficacy of therapeutic massage in conjunction with conventional therapy for children with FD, yet systematic reviews on this topic remain scarce. A systematic review exists that examined therapeutic massage and its combination with conventional therapy for patients with FD. However, this study encompassed individuals of all ages and did not specifically target the pediatric population (Dai et al., 2023). Therefore, we conducted an updated systematic review and meta-analysis to assess the efficacy of therapeutic massage in conjunction with conventional therapy for children with FD.

## 2 Methods

The protocol of this review was registered in the PROSPERO database (2024: CRD42024540844) and this review followed the Preferred Reporting Items for Systematic Reviews and Meta-Analysis (PRISMA) 2020 guidelines. All studies included in this systematic review and meta-analysis were reported to have obtained ethical approval and informed consent from participants, in compliance with the Declaration of Helsinki.

### 2.1 Search strategy

We conducted a search of electronic databases, including the China National Knowledge Infrastructure (CNKI), Wanfang, China Scientific Journals Database (VIP), SinoMed, PubMed, Cochrane Library, Embase, Web of Science, and OVID, for relevant articles published in Chinese or English from inception to November 26, 2024. Additionally, we searched https://clinicaltrials.gov for unpublished trials or supplementary data pertaining to potentially eligible studies. We developed the retrieval strategy using a combination of MeSH and free terms, in consultation with librarians. Boolean operators (AND, OR, NOT, AND NOT) were employed as conjunctions to combine or exclude search terms during the search process. The Mesh terms utilized include “Massage” (按摩), child (儿童), dyspepsia (消化不良), along with free terms such as “Tuina” (推拿), “therapeutic massage,” “children,” functional dyspepsia (功能性消化不良), and “Indigestion.” We developed various retrieval strategies tailored to specific databases. The search strategies from PubMed are presented as follows.

#1 ((Child[MeSH Terms])) OR (Children[Title/Abstract])

#2((((massage[MeSH Terms]) OR (Massage Therapy[Title/Abstract])) OR (Massage Therapies[Title/Abstract])) OR (Therapies, Massage[Title/Abstract])) OR (Therapy, Massage[Title/Abstract])

#3 ((((Dyspepsia[MeSH Terms]) OR (Dyspepsias[Title/Abstract])) OR (Indigestion[Title/Abstract])) OR (Indigestions[Title/Abstract])) OR (functional dyspepsia[Title/Abstract])

### 2.2 Inclusion and exclusion criteria

#### 2.2.1 Type of studies

Randomized controlled trials (RCTs) were included. While other studies, such as case reports, animal research, and literature reviews, were excluded. Conference abstracts were excluded if additional details could not be obtained from the authors.

#### 2.2.2 Type of participants

Children (participants aged <18 years old) with diagnosed FD were included. Diagnostic criteria were as follows: Rome Ⅲ criteria; 2010 Edition Consensus opinion on integrated Chinese and Western medicine in the diagnosis and treatment of functional dyspepsia; Rome Ⅳ criteria; 2017 Edition Diagnosis and treatment plan of Chinese medicine in the stomach (functional dyspepsia); Guidelines for Diagnosis and Treatment of Common Diseases of Pediatries in Traditional Chinese Medicine.

#### 2.2.3 Type of interventions

The experimental intervention was therapeutic massage combined with conventional therapy. We did not limit the manipulation technique of the therapy massage, the number of therapeutic massage sessions, or the duration of the therapy massage. Conventional therapy includes pharmacotherapy, diet care, regular dietary guidance, complementary and alternative therapies, etc.

#### 2.2.4 Type of outcome measures

##### 2.2.4.1 Primary outcome

The primary outcomes were the overall response rate and the symptom scores.

##### 2.2.4.2 Secondary outcome

The secondary outcomes included the levels of growth hormone-releasing peptide, neuropeptide Y, leptin, motilin, gastrin, and 5-hydroxytryptophan in the patients.

### 2.3 Study selection and data extraction

Two researchers independently screened the retrieved studies according to the inclusion and exclusion criteria. If disagreements occurred, the researchers discussed the eligibility of the disputed study with a third researcher until a consensus was reached. Information was extracted from the selected studies using a self-designed data collection form that included the following: information on publication and study characteristics, such as diagnostic criteria, sample size, interventions in the control and experimental groups, duration, adverse events, and outcomes.

### 2.4 Risk of bias

We evaluated the risk of bias in the selected studies using the Cochrane Handbook for Systematic Reviews of Interventions version 5.1.0.20. The risk of bias evaluation involves seven domains: random sequence generation, allocation concealment, blinding of researchers and participants, blinding of evaluators, completeness of outcome indicators, selective reporting, and other bias. The risk of bias in each study was assessed independently by two researchers, who subsequently verified their evaluations with one another. In the absence of a consensus, a third researcher was invited to participate in the evaluation. The findings were categorized as high, low, or unclear risk of bias.

### 2.5 Evaluation of the quality of the evidence body

The Grading of Recommendations, Assessment, Development, and Evaluation (GRADE) framework was employed to assess the quality of the evidence base. The GRADE approach (GRADEpro GDT software, McMaster University, and Evidence Prime Inc.) categorizes the quality of evidence for each outcome into four levels: high, moderate, low, and very low. Two researchers, Lin SH and Lin Y, conducted independent assessments of the evidence quality. In the absence of consensus, disagreements were addressed through discussion, or the final decision was made by the two experienced review authors, Huang XH and Wu GH.

### 2.6 Statistical analysis

The data analysis was conducted using Cochrane Review Manager version 5.4 software. Measurement data were reported as mean differences (MDs), while count data were expressed as odds ratios (ORs). Studies were deemed homogeneous when I^2^ ≤ 50%, leading to the application of the fixed effect model; conversely, when significant heterogeneity was present, the random effect model was utilized. A subgroup analysis or sensitivity analysis was conducted, and potential reasons for the high heterogeneity were identified. A funnel chart was employed to assess publication bias when the analysis included more than 10 studies. Statistical significance was determined at *P* < 0.05.

## 3 Result

### 3.1 Searching and screening

**FIGURE 1:**
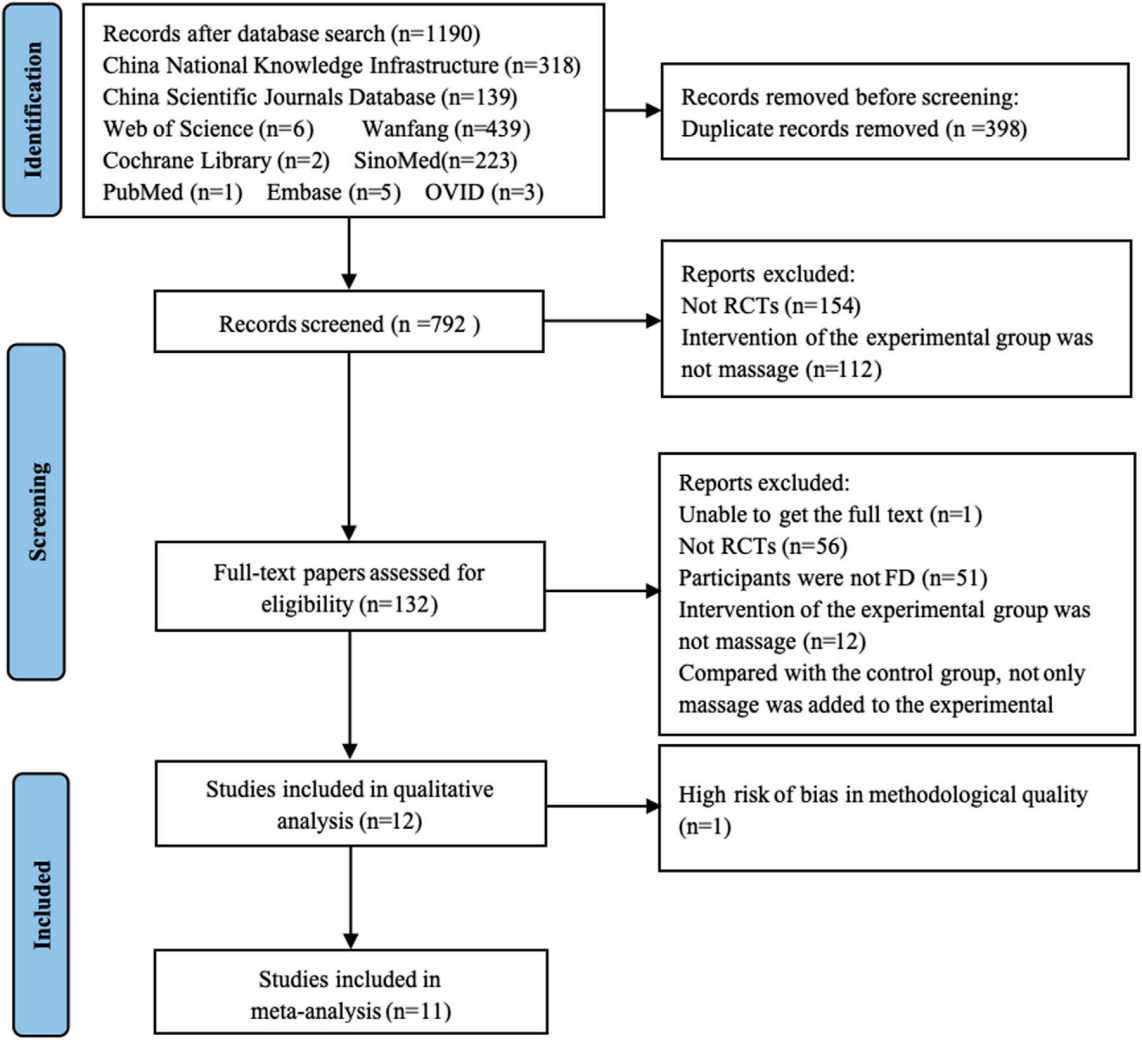
Flowchart of the systematic review and meta-analysis.

From nine databases, 1,190 records were obtained. Finally, twelve trials met the included criteria and were used for system review and meta-analyses. The flow diagram is shown in Figure 1.

### 3.2 Study characteristics

Table 1 shows the characteristics of the twelve RCTs (Yu et al., 2024; Cui and Zhao, 2024; Cao et al., 2024; Dong et al., 2023; Huang, 2022; Zhou et al., 2020; Liu et al., 2020; Xu et al., 2019; Lu and Zhang, 2019; Sheng, 2019; Yang, 2019; Zhou et al., 2019) published between 2019 and 2024, including 1,161 participants. All RCTs were published in Chinese and compared therapeutic massage combined with conventional therapy against conventional therapy alone. The duration of therapeutic massage varied across studies: 2 weeks in seven studies (Cui and Zhao, 2024; Dong et al., 2023; Huang, 2022; Zhou et al., 2020; Lu and Zhang, 2019; Sheng, 2019; Zhou et al., 2019), 4 weeks in three studies (Cao et al., 2024; Xu et al., 2019; Yang, 2019), 6 weeks in one study (Yu et al., 2024) and one study (Liu et al., 2020) did not report the duration of therapeutic massage. Seven RCTs reported overall response rate (Cao et al., 2024; Zhou et al., 2020; Liu et al., 2020; Lu and Zhang, 2019; Sheng, 2019; Yang, 2019; Zhou et al., 2019), six RCTs (Cao et al., 2024; Dong et al., 2023; Zhou et al., 2020; Liu et al., 2020; Lu and Zhang, 2019; Sheng, 2019) provided data on symptom scores, while only one RCT (Yang, 2019) reported the total symptoms score. Eight RCTs (Yu et al., 2024; Cui and Zhao, 2024; Dong et al., 2023; Huang, 2022; Zhou et al., 2020; Lu and Zhang, 2019; Yang, 2019; Zhou et al., 2019) reported gastrointestinal hormones, including growth hormone-releasing peptide, neuropeptide Y, Leptin, motlin, gastrin, plasma substance P, 5-hydroxytryptophan. Additionally, two RCTs provided data on the adverse reaction rate.

**TABLE 1:** Basic characteristics of the included studies.

Studies and year	Diagnostic criteria	Age (years)	Total patients (E/C)	Intervention		Frequency (per week)	Duration (weeks)	Therapeutic massage method	Outcome
				E	C				
Yu ST 2024	②	E:6.61 ± 0.73 C:6.58 ± 0.76	106 (53/53)	Infantile Massage+therapeutic massage	Infantile Massage	1 times × 3 days	6	Point massage	③⑥⑦
Cui XL 2024	②	E:7.46 ± 2.71 C:8.76 ± 2.47	80 (40/40)	Acupoint application+therapeutic massage	Acupoint application	1 time × 6 days	2	Point massage	⑦③
Cao RH 2024	⑤	E:24.93 ± 2.70 C:25.80 ± 2.52 (month)	65 (32/33)	Conventional therapy+therapeutic massage	Conventional therapy	1 time × 5 days	4	Point massage +Chiropractic	①④
Dong Y 2023	①②	E:4.67±0.83 C:4.82±0.74	68 (34/34)	Jianweixiaoshi oral liquid+therapeutic massage	Jianweixiaoshi oral liquid	1 time × 6 days	2	Point massage	①③⑤
Huang QR 2022	③	E:4.28±0.95 C:4.12±0.89	122(61/61)	Acupoint application+therapeutic massage	Acupoint application	1 time × 7 days	2	Point massage	③⑧
Zhou HY 2020	①②	E:5.11 ± 0.76 C:5.12 ± 0.82	100 (50/50)	Basic diet care+therapeutic massage	Basic diet care	1 time × 7 days	2	Chiropractic	①③④
Liu RY 2020	Not report	E:1.67 ± 0.31 C:1.64 ± 0.26	52 (26/26)	Regular dietary guidance+psychological care+regulation of intestinal flora+Jianweixiaoshi oral liquid+therapeutic massage	Regular dietary guidance+psychological care+regulation of intestinal flora+Jianweixiaoshi oral liquid	Not report	Not report	Point massage	①④
Xu JH 2019	①	E:6.28 ± 2.24 C:6.72±2.33	60 (30/30)	Acupoint application+therapeutic massage	Acupoint application	1 time × 5 days	4	Chiropractic	
Lu T 2019	①	E:7.23 ± 0.81 C:7.31 ± 0.76	248 (124/124)	Xingpi Yang'er Granules+therapeutic massage	Xingpi Yang'er Granules	1time × 5 days	2	Point massage	①③④
Shen LX 2019	①	E:7.30±0.90 C:6.90±1.10	126 (63/63)	Conventional therapy+therapeutic massage	Conventional therapy	1 time × 5 days	2	Point massage	①④
Yang Y 2019	③④	E:4.67 ± 0.83 C:5.80 ± 2.00	84 (42/42)	Gastro-kinetic agent+Gastric mucosal protective drugs+Si Mo Tang Oral Liquid+therapeutic massage	Gastro-kinetic agent+Gastric mucosal protective drugs+Si Mo Tang Oral Liquid	2 times × 7 days	4	Point massage	②③④⑤
Zhou L 2019	③	E:5.80 ± 2.80 C:6.10 ± 2.70	50 (25/25)	Omeprazole capsules+therapeutic massage	Omeprazole capsules	1 time × 7 days	2	Point massage	③④

**Note:** C, Control group; E, Experiment group (Massage group).

**Diagnostic criteria:** ①Rome III criteria; ②2010 Edition “Consensus opinion on integrated Chinese and Western medicine in the diagnosis and treatment of functional dyspepsia”; ③Rome Ⅳ criteria; ④2017 Edition “stuffiness of stomach (Functional Dyspepsia) Chinese Medicine Diagnosis and Treatment Plan”; ⑤Guidelines for Diagnosis and Treatment of Commom Diseases of Pediatries in Traditional Chinese Medicine.

**Outcome:** ①Symptom scores, including one or more of the following symptoms: abdominal pain, flatulence, anorexia, eructation, nausea and vomiting, discomfort of early satiety; ②Total Symptom Score; ③ Gastrointestinal hormones, such as growth hormone-releasing peptide (GHRP), Neuropeptide Y(NPY), Leptin, motlin, gastrin, Plasma substance P, 5-Hydroxytryptophan, and so on; ④overall response rate; ⑤adverse reaction rate; ⑥Nepean Dyspepsia Index; ⑦Traditional Chinese Medicine Symptom scores; ⑧Symptom resolution time.

### 3.3 Risk of bias in included studies

#### 3.3.1 Random sequence generation

Seven RCTs (Yu et al., 2024; Cui and Zhao, 2024; Cao et al., 2024; Dong et al., 2023; Huang, 2022; Zhou et al., 2020; Yang, 2019) that utilized random number tables were classified as having a low risk of bias. Five RCTs (Liu et al., 2020; Xu et al., 2019; Lu and Zhang, 2019; Sheng, 2019; Zhou et al., 2019) that failed to report their methods of random sequence generation were deemed to have an unclear risk of bias.

#### 3.3.2 Allocation concealment

None of the RCTs reported allocation concealment, and therefore were all assessed as unclear risk of bias.

#### 3.3.3 Blinding of participants and personnel

Since none of the RCTs reported blinding of participants and personnel, they were all rated as having an unclear risk of bias.

#### 3.3.4 Blinding of outcome assessment

Twelve RCTs were assessed as unclear risk of bias because none of them reported blinding of outcome evaluation.

#### 3.3.5 Incomplete outcome data

All RCTs without missing participants were evaluated as low risk of bias.

#### 3.3.6 Selective reporting

Twelve RCTs that reported results as described in the methods were evaluated as low risk of bias.

#### 3.3.7 Other bias

**FIGURE 2:**
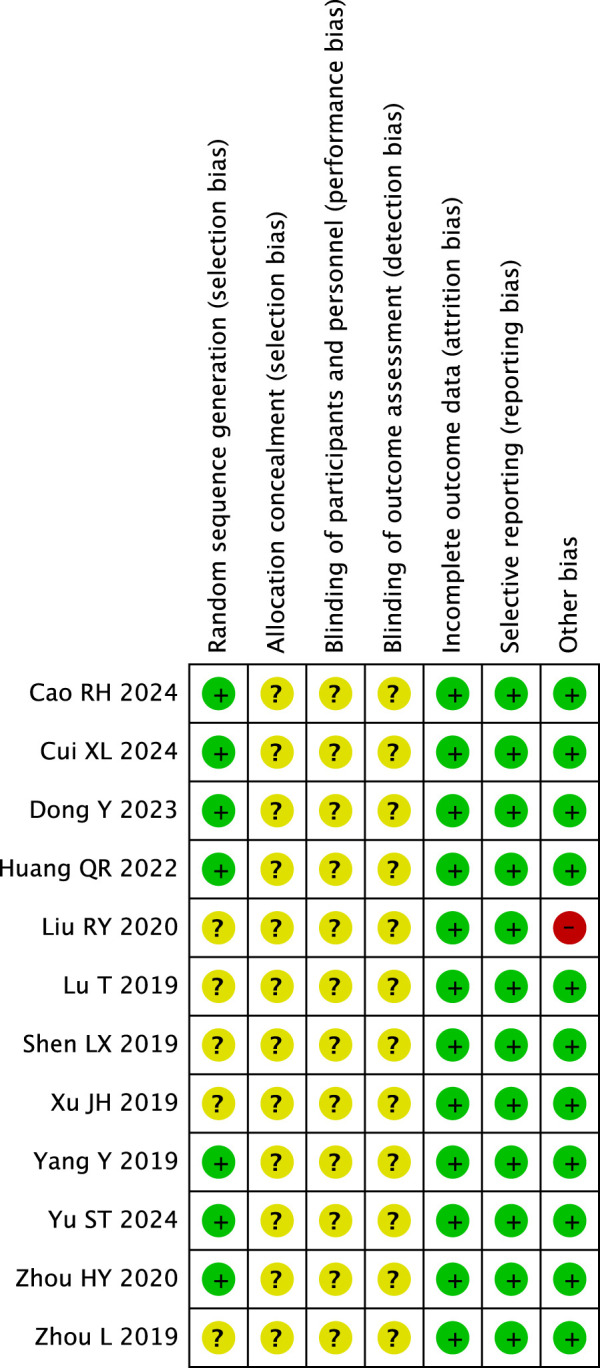
Risk-of-bias summary.

Eleven RCTs received government funding, which did not appear to influence the risk of bias. One RCT (Liu et al., 2020) that did not disclose the frequency of therapeutic massage was evaluated as having a high risk of bias. Figure 2 illustrates the bias risk associated with the 12 randomized controlled trials (RCTs).

### 3.4 Meta-analysis

Twelve studies were included, of which eleven studies were merged in the meta-analysis.

#### 3.4.1 The overall response rate

**FIGURE 3:**
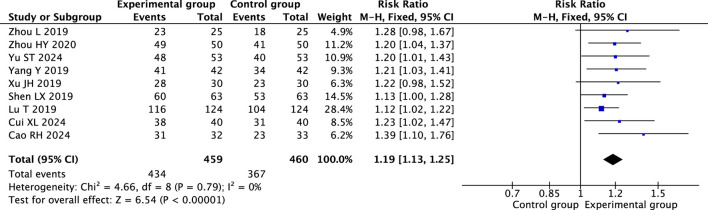
Forest plot comparing the efficacy of therapeutic massage combined with conventional therapy (TMC) versus conventional therapy alone (CT) on overall response rate in pediatric patients with functional dyspepsia (FD). TMC demonstrated a significantly higher overall response rate compared to CT alone (pooled RR = 1.19, 95% CI: 1.13–1.25, *P* < 0.001, I^2^ = 0%).

**FIGURE 4:**
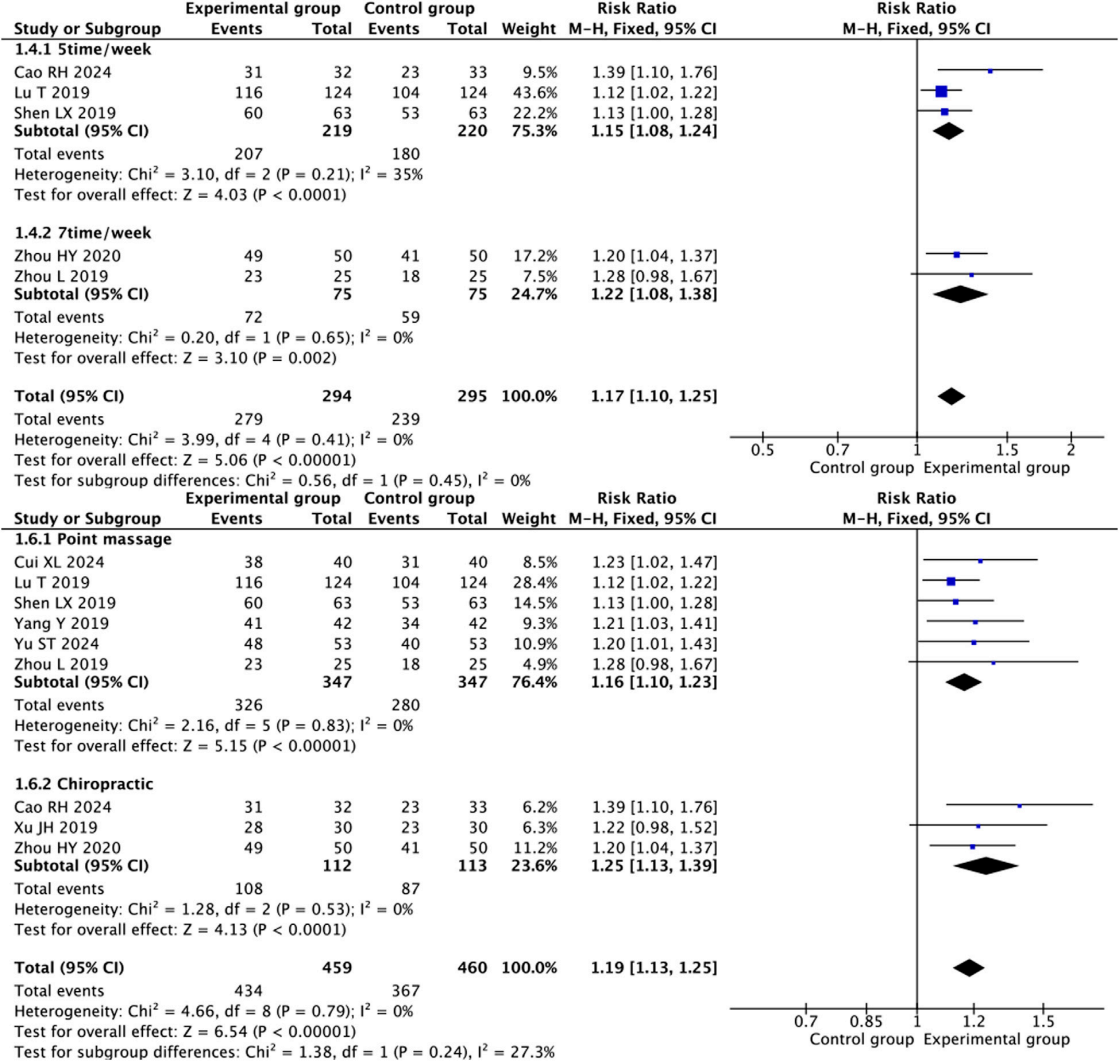
Forest plot illustrating the efficacy of therapeutic massage combined with conventional therapy (TMC) versus conventional therapy (CT) on overall response rate in pediatric patients with functional dyspepsia (FD): subgroup analysis by frequency and type of intervention. Overall response rates were significantly higher for TMC compared to CT alone in both subgroup analyses of intervention frequency (pooled RR = 1.17, 95% CI: 1.10–1.25, *P* < 0.001, I^2^ = 0%) and subgroup analyses of intervention modality (pooled RR = 1.19, 95% CI: 1.13–1.25, *P* < 0.001, I^2^ = 0%).

Nine RCTs (Yu et al., 2024; Cui and Zhao, 2024; Cao et al., 2024; Zhou et al., 2020; Xu et al., 2019; Lu and Zhang, 2019; Sheng, 2019; Yang, 2019; Zhou et al., 2019) showed that therapeutic massage combined with conventional therapy had a better effect on the overall response rate than conventional therapy (RR = 1.19; 95% CI 1.13 to 1.25, I^2^ = 0%, *P* < 0.001; Figure 3). A subgroup analysis based on the frequency of therapeutic massage indicated that both five times per week (Cao et al., 2024; Lu and Zhang, 2019; Sheng, 2019) (RR = 1.15; 95% CI 1.08 to 1.24, I^2^ = 35%, *P* < 0.001; Figure 4) and seven times per week (Zhou et al., 2020; Zhou et al., 2019) (RR = 1.22; 95% CI 1.08 to 1.38, I^2^ = 0%, *P* = 0.002; Figure 4) in conjunction with conventional therapy resulted in a higher overall response rate compared to conventional therapy alone in children with FD. A subgroup analysis based on the type of therapeutic massage indicated that both point massage (Yu et al., 2024; Cui and Zhao, 2024; Lu and Zhang, 2019; Sheng, 2019; Yang, 2019; Zhou et al., 2019) (RR = 1.16; 95% CI 1.10 to 1.23, I^2^ = 0%, *P* < 0.001; Figure 4) and chiropractic (Cao et al., 2024; Zhou et al., 2020; Xu et al., 2019) (RR = 1.25; 95% CI 1.13 to 1.39, I^2^ = 0%, *P* < 0.001; Figure 4) in combination with conventional therapy resulted in a higher overall response rate compared to conventional therapy alone in children with FD.

#### 3.4.2 Symptom scores

**FIGURE 5:**
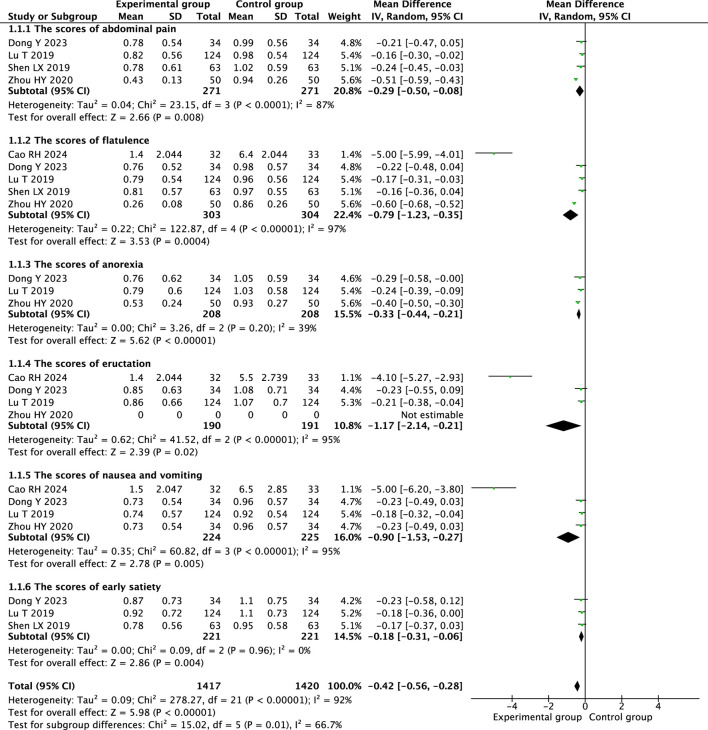
Forest plot comparing the efficacy of therapeutic massage combined with conventional therapy (TMC) versus conventional therapy (CT) alone on symptom scores in pediatric patients with functional dyspepsia (FD). TMC decrease the symptom scores compared to CT alone (pooled MD = −0.42 95% CI: −0.56 to −0.28, *P* < 0.001, I^2^ = 92%).

**FIGURE 6:**
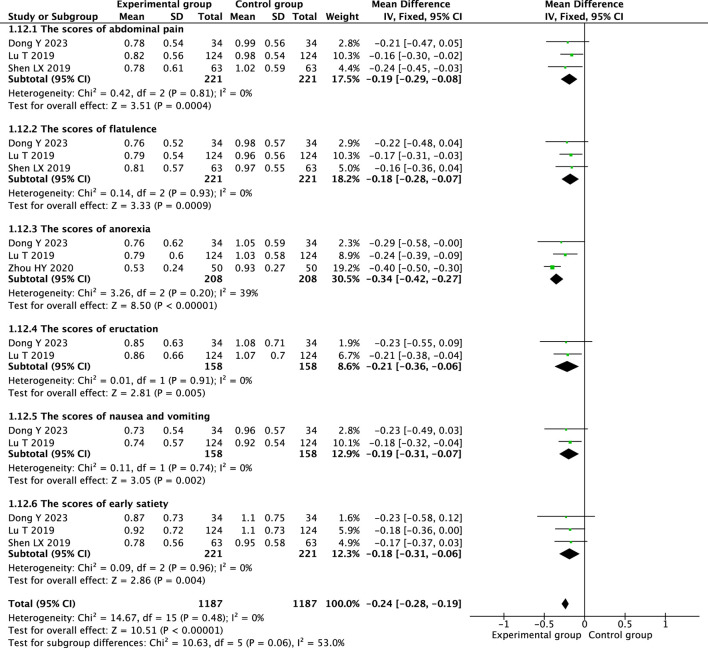
Sensitivity analysis forest plot illustrating the symptom scores of therapeutic massage combined with conventional therapy (TMC) versus conventional therapy (CT) in pediatric patients with functional dyspepsia (FD) after excluding high-risk-of-bias studies. TMC decrease the symptom scores compared to CT alone (pooled MD = −0.24 95% CI: −0.28 to −0.19, *P* < 0.001, I^2^ = 0%).

Therapeutic massage combined with conventional therapy showed a better effect on relieving abdominal pain (Dong et al., 2023; Zhou et al., 2020; Lu and Zhang, 2019; Sheng, 2019) (MD = −0.29; 95% CI −0.50 to −0.08, I^2^ = 87%, *P* = 0.008; Figure 5), but the heterogeneity is high. A heterogeneity sensitivity analysis for heterogeneity was performed after excluding the RCT by Zhou HY (Zhou et al., 2020), and the subsequent meta-analysis yielded the following results (MD = −0.19; 95% CI 0.29 to −0.08, I^2^ = 0%, *P* < 0.001; Figure 6). The result showed that the heterogeneity may be attributable to differences in the intervening measure.

Therapeutic massage combined with conventional therapy demonstrated a more significant impact on alleviating flatulence (Cao et al., 2024; Dong et al., 2023; Zhou et al., 2020; Lu and Zhang, 2019; Sheng, 2019) (MD = −0.79; 95% CI −1.23 to −0.35, I^2^ = 97%, *P* = 0.0004; Figure 5), although the heterogeneity is considerable. A sensitivity analysis addressing heterogeneity was conducted following the exclusion of the RCTs by Cao RH (Cao et al., 2024) and Zhou HY (Zhou et al., 2020), the resulting meta-analysis produced the following outcomes: (MD = −0.18; 95% CI -0.28 to −0.07, I^2^ = 0%, *P* < 0.001; Figure 6). The results indicate that heterogeneity may be due to the differences in the intervening measure and intervention frequency.

Two RCTs (Dong et al., 2023; Lu and Zhang, 2019) showed that that therapeutic massage, when combined with conventional therapy, significantly reduced the anorexia scores in children with functional dyspepsia compared to conventional therapy alone (MD = −0.34; 95% CI −0.42 to −0.27, I^2^ = 39%, *P* < 0.001; Figure 6).

Therapeutic massage combined with conventional therapy showed a better effect on relieving eructation (Cao et al., 2024; Dong et al., 2023; Zhou et al., 2020; Lu and Zhang, 2019) (MD = −1.17; 95% CI −2.14 to −0.21, I^2^ = 95%, *P* = 0.02; Figure 5), although the heterogeneity remains considerable. A sensitivity analysis for heterogeneity was conducted following the exclusion of the RCTs by Cao RH (Cao et al., 2024) and Zhou HY (Zhou et al., 2020). The resulting meta-analysis produced the following outcomes: (MD = −0.21; 95% CI −0.36 to −0.06, I^2^ = 0%, *P* = 0.005; Figure 6). The results indicated that the heterogeneity could be due to variations in the intervening measure and the duration of the intervention.

Therapeutic massage combined with conventional therapy showed a better effect on relieving nausea and vomiting (Cao et al., 2024; Dong et al., 2023; Zhou et al., 2020; Lu and Zhang, 2019) (MD = −0.90; 95% CI −1.53 to −0.27, I^2^ = 95%, *P* = 0.005; Figure 5), but the heterogeneity is high. A heterogeneity sensitivity analysis for heterogeneity was performed after excluding the RCT by Cao et al. (2024) and Zhou et al. (2020), and the subsequent meta-analysis yielded the following results (MD = −0.19; 95% CI −0.31 to −0.06, I^2^ = 0%, *P* = 0.002; Figure 6). The result showed that the heterogeneity may be attributable to differences in the intervening measure and intervention duration.

Three RCTs (Dong et al., 2023; Lu and Zhang, 2019; Sheng, 2019) showed that compared with conventional therapy, therapeutic massage combined with conventional therapy significantly decreased the score of early satiety of the children with FD (MD = −0.18; 95% CI −0.31 to −0.06, I^2^ = 0%, *P* = 0.004; Figure 6).

#### 3.4.3 Growth hormone-releasing peptide and 5-hydroxytryptophan

**FIGURE 7:**
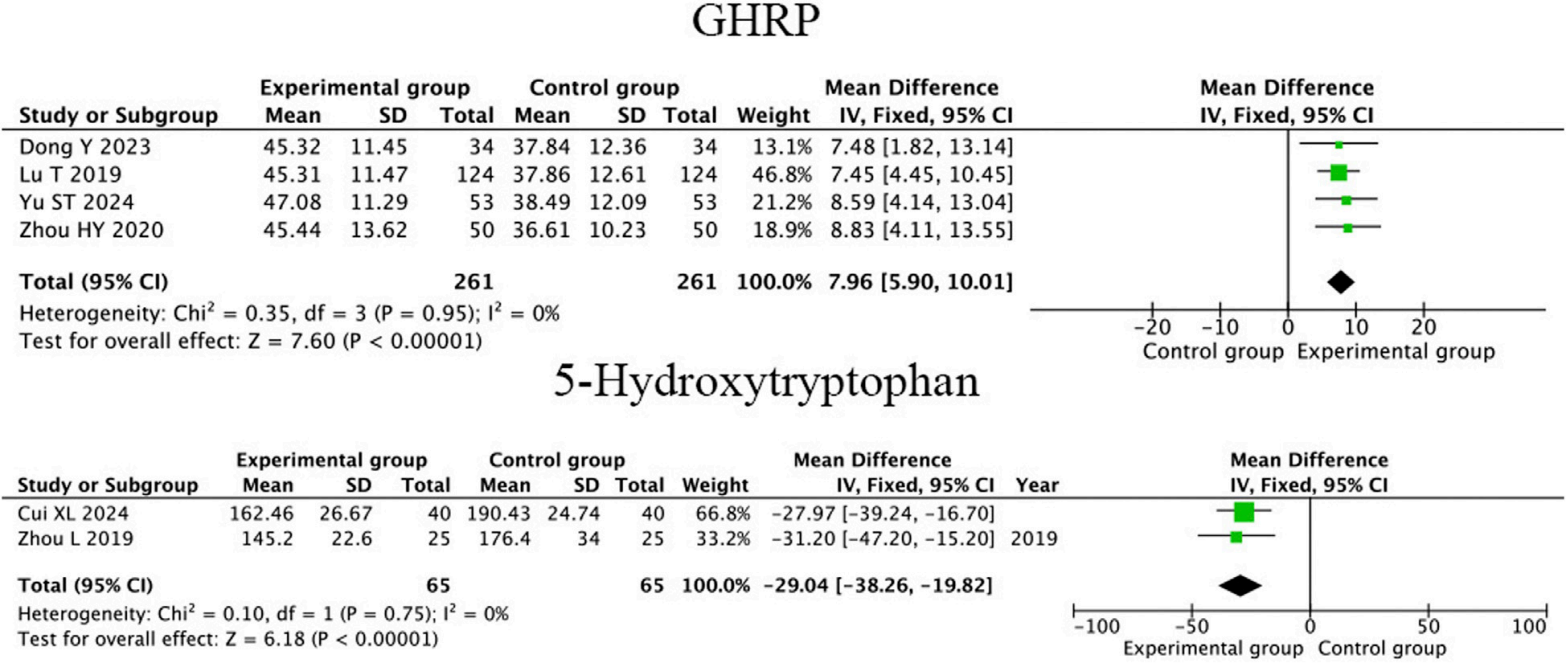
Forest plot comparing the efficacy of therapeutic massage combined with conventional therapy (TMC) versus conventional therapy alone (CT) on serum GHRP and 5-HT levels in pediatric patients with functional dyspepsia (FD). TMC decrease the level of serum GHRP (pooled MD = 7.96, 95% CI: 5.90–10.01, *P* < 0.001, I^2^ = 0%). And 5-HT levels (pooled MD = -29.04, 95% CI: −38.26–19.82, *P* < 0.001, I^2^ = 0%) compared to CT alone.

Four RCTs (Yu et al., 2024; Dong et al., 2023; Zhou et al., 2020; Lu and Zhang, 2019) that therapeutic massage, when combined with conventional therapy, significantly elevated the GHRP levels in children with FD compared to conventional therapy alone (MD = 7.96; 95% CI 5.90 to 10.01, I^2^ = 0%, *P* < 0.001; Figure 7). Two RCTs (Cui and Zhao, 2024; Zhou et al., 2019) that therapeutic massage, when combined with conventional therapy, significantly reduced the 5-hydroxytryptophan levels in children with functional dyspepsia compared to conventional therapy alone (MD = −29.04; 95% CI −38.26 to −19.82, I^2^ = 0%, *P* < 0.001; Figure 7).

#### 3.4.4 Neuropeptide Y, leptin, motolin and gastrin

**FIGURE 8:**
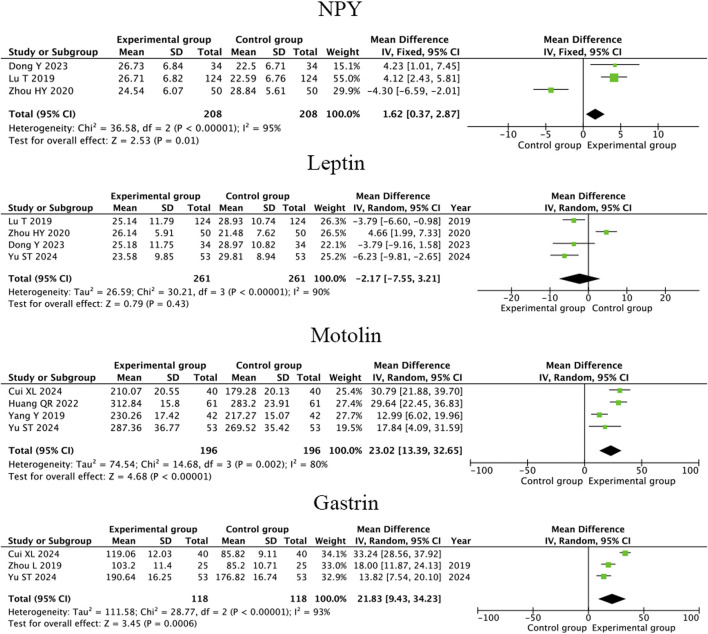
Forest plot comparing the efficacy of therapeutic massage combined with conventional therapy (TMC) versus conventional therapy alone (CT) on serum NPY,leptin, motolin and gastrin levels in pediatric patients with functional dyspepsia (FD). TMC improve the level of serum NPY (pooled MD = 1.62, 95% CI: 0.37–2.87, *P* < 0.001, I^2^ = 95%), motolin (pooled MD = 23.02, 95% CI: 13.39–32.65, *P* < 0.001, I^2^ = 80%) and gastrin (pooled MD = 21.83, 95% CI: 9.43–34.23, *P* < 0.001, I^2^ = 93%), decrease the level of leptin (pooled MD = −2.17, 95% CI: −7.55–3.21, *P* < 0.001, I^2^ = 90%) compared to CT alone.

**FIGURE 9:**
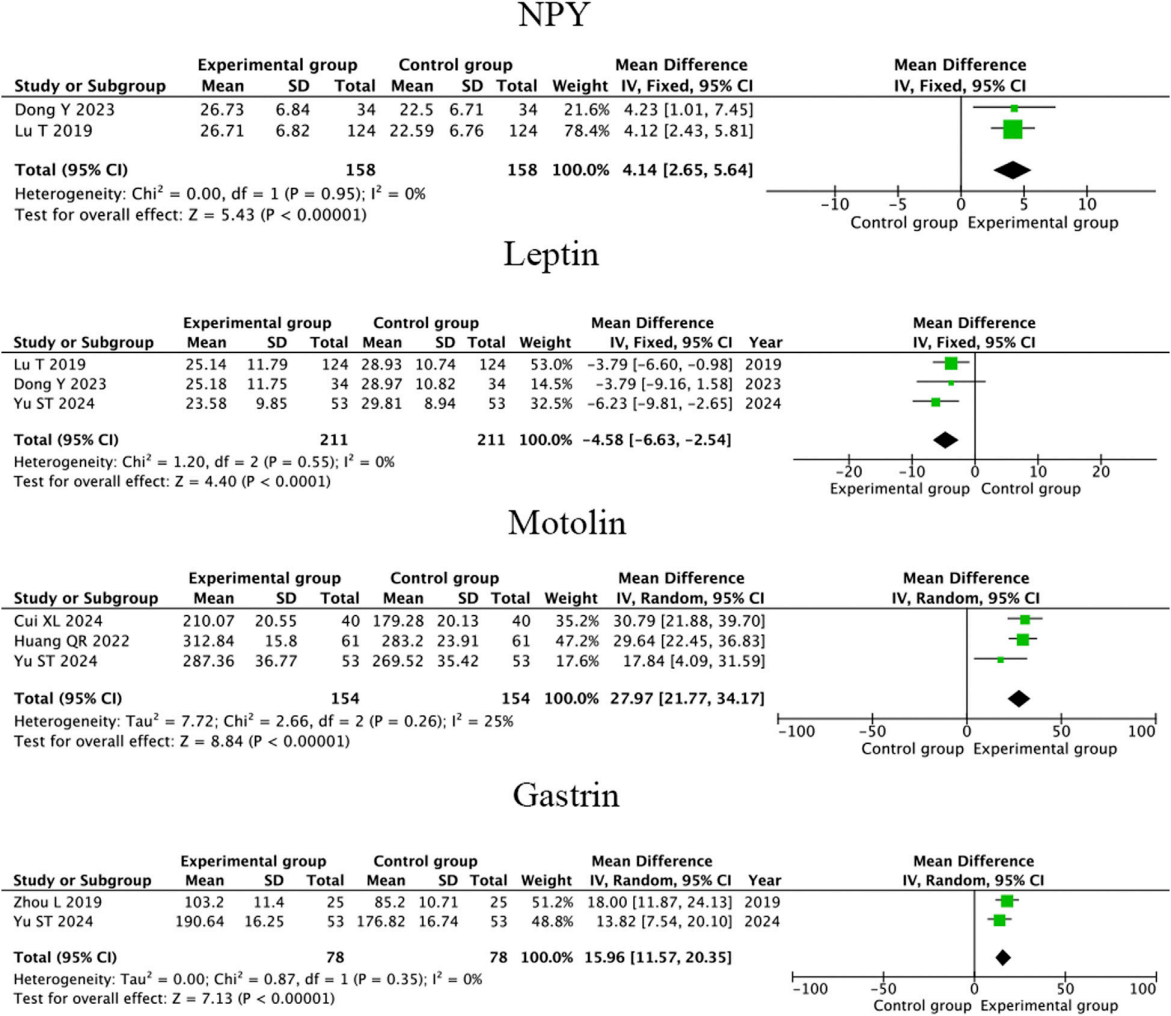
Sensitivity analysis forest plot illustrating the comparative effects of therapeutic massage combined with conventional therapy (TCM) versus conventional therapy (CT) on serum NPY, leptin, motolin and gastrin levels in pediatric patients with FD after excluding high-risk-of-bias studies. TMC improve the level of serum NPY (pooled MD = 4.14, 95% CI: 2.65–5.64, *P* < 0.001, I^2^ = 0%), motolin (pooled MD = 27.97, 95% CI: 21.77–34.17, *P* < 0.001, I^2^ = 25%) and gastrin (pooled MD = 15.96, 95% CI: 11.57–20.35, *P* < 0.001, I^2^ = 0%), decrease the level of leptin (pooled MD = -4.58, 95% CI: −6.63 to −2.54, *P* < 0.001, I^2^ = 0%) compared to CT alone.

Three RCTs (Dong et al., 2023; Zhou et al., 2020; Lu and Zhang, 2019) showed that compared to conventional therapy, therapeutic massage combined with conventional therapy significantly increased the level of NPY in children with FD (MD = 1.62; 95% CI 0.37 to 2.87, I^2^ = 95%, *P* = 0.01; Figure 8) but the heterogeneity is high. A heterogeneity sensitivity analysis for heterogeneity was performed after excluding the RCT by Zhou et al. (2020), and the subsequent meta-analysis yielded the following results (MD = 4.14; 95% CI 2.65 to 5.64, I^2^ = 0%, *P* < 0.001; Figure 9). The result showed that the heterogeneity may be attributable to differences in the intervening measure.

Four RCTs (Yu et al., 2024; Dong et al., 2023; Zhou et al., 2020; Lu and Zhang, 2019) showed that compared to conventional therapy, therapeutic massage combined with conventional therapy has no significant difference in increasing the level of Leptin in children with FD (MD = −2.17; 95% CI −7.55 to 3.21 I^2^ = 90%, *P* = 0.43; Figure 8) and the heterogeneity is high. A heterogeneity sensitivity analysis for heterogeneity was performed after excluding the RCT by Zhou et al. (2020), and the subsequent meta-analysis yielded that compared to conventional therapy, therapeutic massage combined with conventional therapy significantly increased the level of Leptin in FD children (MD = −4.58; 95% CI −6.63 to −2.54, I^2^ = 0%, *P* < 0.001; Figure 9). The result showed that the heterogeneity may be attributable to differences in the intervening measure.

Four RCTs (Yu et al., 2024; Cui and Zhao, 2024; Huang, 2022; Yang, 2019) showed that compared to conventional therapy, therapeutic massage combined with conventional therapy significantly increased the level of Motolin in children with FD (MD = 23.03; 95% CI 13.39 to 32.65, I^2^ = 80%, *P* < 0.001; Figure 8) but the heterogeneity is high. A heterogeneity sensitivity analysis for heterogeneity was performed after excluding the RCT by Yang (2019), and the subsequent meta-analysis yielded the following results (MD = 27.97; 95% CI 21.77 to 34.17 I^2^ = 25%, *P* < 0.001; Figure 9). The result showed that the heterogeneity may be attributable to differences in the intervention duration.

Three RCTs (Yu et al., 2024; Cui and Zhao, 2024; Zhou et al., 2019) showed that compared to conventional therapy, therapeutic massage combined with conventional therapy significantly increased the level of gastrin in children with FD (MD = 21.83; 95% CI 9.43 to 34.23, I^2^ = 93%, *P* < 0.001; Figure 8) but the heterogeneity is high. A heterogeneity sensitivity analysis for heterogeneity was performed after excluding the RCT by Cui and Zhao (2024), and the subsequent meta-analysis yielded the following results (MD = 15.96; 95% CI 11.57 to 20.35 I^2^ = 0%, *P* < 0.001; Figure 9). The result showed that the heterogeneity may be attributable to differences in the intervention duration.

#### 3.4.5 Safety of therapeutic massage combined with conventional therapy for FD

One study indicated the safety of therapeutic massage in conjunction with conventional therapy for children with FD. The findings revealed a lower incidence of adverse effects in children receiving therapeutic massage alongside conventional therapy compared to those undergoing only conventional therapy (*X* ^2^ = 10.592, *P* < 0.05). Other studies did not report adverse events, indicating the relative safety of therapeutic massage.

### 3.5 The overall quality of evidence

The evidence quality for all outcomes varied from moderate to very low. The low ratings were attributed to the absence of allocation concealment, insufficient sample sizes, and reliance on surrogate outcomes. Table 2 presents the evidence quality for each result.

**TABLE 2:** GRADE quality grading evaluation of the outcomes.

Certainty assessment							Summarry of findings			
Outcome (No. of studies)	Study design	Risk of bias	Inconsistency	Indirectness	Imprecision	Other considerations	No. of patients		Effect	Certainty
							intervention	comparison	Absolute (95% CI)	
Overall response rate (9)	RCTs	serious^a^	not serious	not serious	not serious	none	459	460	128 more per 1,000 (from 72 more to 183 more)	⊕⊕⊕⊖ Moderate
Overall response rate(5times/week) (3)	RCTs	serious^a^	not serious	not serious	serious	none	219	220	98 more per 1,000 (from 33 more to 164 more)	⊕⊕⊕⊖ Moderate
Overall response rate(7times/week) (2)	RCTs	serious^a^	not serious	not serious	serious^b^	none	75	75	173 more per 1,000 (from 63 more to 299 more)	⊕⊕⊖⊖ Low
The scores of abdominal pain (3)	RCTs	serious^a^	not serious	not serious	not serious	none	221	221	MD 0.19 lower (0.29 lower to 0.08 lower)	⊕⊕⊕⊖ Moderate
The scores of flatulence (3)	RCTs	serious^a^	not serious	not serious	not serious	none	221	221	MD 0.18 lower (0.28 lower to 0.07 lower)	⊕⊕⊕⊖ Moderate
The scores of anorexia (2)	RCTs	serious^a^	not serious	not serious	serious^b^	none	158	158	MD 0.25 lower (0.38 lower to 0.12 lower)	⊕⊕⊖⊖ Low
The scores of eructation (2)	RCTs	serious^a^	not serious	not serious	serious^b^	none	158	158	MD 0.21 lower (0.36 lower to 0.06 lower)	⊕⊕⊖⊖ Low
The scores of nausea and vomiting (2)	RCTs	serious^a^	not serious	not serious	serious^b^	none	158	158	MD 0.19 lower (0.31 lower to 0.07 lower)	⊕⊕⊖⊖ Low
The scores of early satiety (3)	RCTs	serious^a^	not serious	not serious	not serious	none	221	221	MD 0.18 lower (0.31 lower to 0.06 lower)	⊕⊕⊕⊖ Moderate
NPY (2)	RCTs	serious^a^	not serious	serious^c^	serious^b^	none	158	158	MD 4.14 higher (2.65 higher to 5.64 higher)	⊕⊖⊖⊖ Very low
GHRP (3)	RCTs	serious^a^	not serious	serious^c^	serious	none	261	261	MD 7.96 higher (5.90 higher to 10.01 higher)	⊕⊕⊖⊖ Low
Leptin (3)	RCTs	serious^a^	not serious	serious^c^	serious	none	211	211	MD -4.58 higher (-6.63 higher to -2.54 higher)	⊕⊕⊖⊖ Low
Motolin (3)	RCTs	serious^a^	not serious	serious^c^	serious^b^	none	154	154	MD 27.97 lower (21.77 lower to 34.17 lower)	⊕⊖⊖⊖ Very low
Gastrin (2)	RCTs	serious^a^	not serious	serious^c^	serious^b^	none	78	78	MD 15.96 lower (11.57 lower to 20.35 lower)	⊕⊖⊖⊖ Very low
5-Hydroxytryptophan (2)	RCTs	serious^a^	not serious	serious^c^	serious^b^	none	65	65	MD -29.04 lower (-38.26 lower to -19.82 lower)	⊕⊖⊖⊖ Very low

^a^ Did not report allocation concealment.

^b^ Lack of sufficient sample size.

^c^ Using of surrogate outcomes.

GRADE, the Grading of Recommendations, Assessment, Development and Evaluation; RCTs, Randomized controlled trials; NPY, Neuropeptide Y; GHRP, growth hormone-releasing peptide.

High certainty: We are very confident that the true effect is close to the estimated effect.

Moderate certainty: We are moderately confident about the estimated effect; the true effect could be close to the estimated effect, but it could be substantially different as well.

Low certainty: We have limited confidence about the estimated effect; the true effect may be substantially different from the estimated effect.

Very low certainty: We have very little confidence in the estimated effect: the true effect is likely to be substantially different from the estimated effect.

## 4 Discussion

### 4.1 Summary of the main findings

This review systematically evaluated the effectiveness of therapeutic massage combined with conventional therapy in children with FD. Moderate certainty evidence showed that therapeutic massage combined with conventional therapy could be beneficial in improving the overall response rate, the overall response with the intervention of 5 times/week and reducing the scores of abdominal pain, flatulence, and early satiety compared to conventional therapy. Low-certainty evidence showed that therapeutic massage combined with conventional therapy might be beneficial to improve the overall response rate with the intervention frequency of 7 times/week, and reduce the scores of anorexia, the scores of eructation, and the scores of nausea and vomiting and increase the level of GHRP and Leptin. Very low certainty evidence showed that therapeutic massage combined with conventional therapy might be beneficial to increase the level of NPY, motolin gastrin and 5-HT. Nevertheless, the validity of these results is constrained by the moderate-to-low certainty of evidence and critical methodological biases. High-quality RCTs with robust allocation concealment and intention-to-treat analysis are urgently needed to confirm clinical benefits.

### 4.2 Compared with previous studies

A comparable article on this subject exists (Dai et al., 2023), which examined FD patients across all age groups and demonstrated that therapeutic massage markedly enhanced the overall symptoms and quality of life for FD patients. In this study, we limited the study population to pediatric FD patients based on the above studies, while gastrointestinal-related hormones were included in terms of outcome metrics, and subgroup analyses were also performed according to the duration of treatment.

There are various ways to categorize therapeutic massage. For example, depending on the subjects, by applied manipulation, therapeutic massage can be divided into therapeutic massage for adults and therapeutic massage for children (pediatric therapeutic massage) (Wang et al., 2024). This review categorises therapeutic massage into two types: point massage and chiropractic, both of which are forms of paediatric therapeutic massage. Point massage is mainly used to massage the acupoints and meridians of the limbs, and the acupoints used in the RCTs included in this review are mainly the Spleen Meridian, Banmen acupoint, Zusanli acupoint, Pishu acupoint, Weishu acupoint, Spleen Meridian, tonifying stomach Meridian and so on. Chiropractic is a traditional Chinese massage that was initially applied for digestion among children by pinching the skin along the spine, and shows very good clinical effects in the treatment of various digestive problems among children (Zhu et al., 2020).

Therapeutic massage belongs to complementary and alternative medicine (CAM), which is therapy designed to promote healing or prevent disease that exists outside of the realm of standard practice. A survey indicated that in Italy, approximately 48.7% of patients with functional dyspepsia utilized CAM alongside standard treatment (Lahner et al., 2013). Additionally, Therapeutic massage is grounded in the principles of traditional Chinese medicine, the practices at the meridian and acupoints, giving the body benign physical stimulation, making the function of zang-fu organs blood of equilibrium state and it is economical, convenient, and safe. This has led to an increase in the use of therapeutic massage in recent years to treat FD.

Satisfactory relief of symptoms is an important objective in the treatment of FD (Vakil et al., 2008). This review indicates that the combination of therapeutic massage and conventional therapy is more effective than conventional therapy alone in alleviating symptoms in children with FD. This effectiveness may stem from the simplicity and painlessness of therapeutic massage, which enhances compliance among children. Additionally, the integration of therapeutic massage with conventional therapy may enhance therapeutic outcomes and mitigate the risk of drug dependency associated with prolonged medication use (Lu and Zhang, 2019).

Approximately 80% of individuals with dyspepsia lack a structural explanation for their symptoms, leading to a diagnosis of functional dyspepsia (Siavosh et al., 2022). The risk factors of FD include psychological comorbidity, acute gastroenteritis, female sex, smoking, use of non-steroidal anti-inflammatory drugs, and *Helicobacter pylori* infection (Ford et al., 2020). The pathophysiology remains incompletely understood, but it is probably related to disordered communication between the gut and the brain, leading to motility disturbances, visceral hypersensitivity, and alterations in gastrointestinal microbiota, mucosal and immune function, and CNS processing. All of these causative mechanisms have the potential to partially explain symptoms in some functional dyspepsia patients, thus providing a rationale for the efficacy of a diversity of therapeutic approaches to functional dyspepsia.

Gastrointestinal dysmotility represents a potential etiology of functional dyspepsia. Gastrointestinal hormones are a group of peptides distributed in the gastrointestinal tract and blood circulation. Some are related to gastrointestinal motility, for instance, GHRP, NPY, leptin, motolin, gastrin, and 5-HT (Liu et al., 2021). Motilin has been confirmed to induce contraction of the smooth muscles in the gastrointestinal tract, thereby enhancing gastrointestinal motility and gastric emptying rate. Gastrin is an acid-stimulatory messenger that can regulate gastric acid secretion and gastric mucosal cell growth, thereby increasing food intake (Li et al., 2022). 5-hydroxytryptamine (5-HT), a metabolite of tryptophan, mediates the development of the enteric nervous system, gut motility, and epithelial development (Yu et al., 2023). Neuropeptide Y is a central facilitator directly related to the feeding factor that transmits hunger signals and increases appetite, similar to the growth hormone-releasing peptide (Lu and Zhang, 2019). Leptin is a neuroendocrine factor produced by the hypothalamus and gastric mucosal cells, which inhibits gastric emptying and promotes satiety (Hammersjo et al., 2016). This review found that therapeutic massage combined with conventional therapy significantly improved the level of GHRP, motolin, leptin, gastrin, NPY, and decrease the level of 5-HT. These findings suggest that therapeutic massage may exert its effects on FD through the regulation of gastrointestinal hormones.

Our findings align with some international studies that have reported positive effects of complementary therapies on FD symptoms, particularly in reducing pain and improving quality of life (Liao et al., 2024). However, differences in study populations, diagnostic criteria, and cultural attitudes toward complementary therapies may explain some variability in results. For example, in Western countries, complementary therapies are often less integrated into mainstream healthcare, which may limit their accessibility and acceptance. In contrast, traditional therapies like massage are more widely accepted and practiced in many Asian cultures.

Furthermore, our findings are consistent with certain aspects of international guidelines, such as the Rome IV criteria (Drossman and Hasler, 2016), which emphasize the importance of a multidisciplinary approach to managing FD. However, international guidelines generally recommend complementary therapies only as adjuncts to conventional treatments, highlighting the need for further research to establish their efficacy and safety (Harvard Health Publishing, 2020).

### 4.3 Recommendation for clinical practice

The inadequate understanding of the pathology and the limited efficacy of conventional treatments for FD have prompted an increasing number of patients to pursue alternative therapies. Therapeutic massage is one such alternative. This review indicates that the combination of therapeutic massage with conventional therapy yields greater benefits in the treatment of FD in children than conventional therapy alone. The treatment regimen from the included trials indicates that children with FD should undergo therapeutic massage for a minimum of 2 weeks, with a frequency of at least five sessions per week. However, the overall quality of the evidence in this paper is rated as low to moderate, which weakens the impact of the findings. While our findings demonstrate the potential benefits of therapeutic massage for FD patients, it is important to acknowledge the heterogeneity observed in the results. This variability may stem from differences in study designs, such as variations in treatment duration, control group selection, and outcome measurement tools. Additionally, cultural differences in therapeutic massage practices, including variations in techniques, intensity, and frequency, could influence treatment outcomes. Inconsistent diagnostic criteria for FD across studies may contribute to heterogeneity by affecting patient selection and subtype classification. The potential impact of these factors on the generalizability of our findings beyond Chinese populations warrants careful consideration. Therapeutic massage practices and healthcare approaches are often shaped by cultural and regional contexts, which may limit the applicability of our results to other populations. For instance, in Western countries, massage therapy may be less integrated into mainstream healthcare, and patient preferences or expectations may differ. Therefore, future methodologically rigorous, multicenter RCTs with adequately powered sample sizes, standardized treatment protocols and outcome measures, systematic documentation of adverse events, and longitudinal follow-up designs are warranted to comprehensively assess the long-term efficacy, safety, and cost-effectiveness of therapeutic massage integrated with conventional therapies in pediatric FD populations.

### 4.4 Recommendations for future research

The decline in evidence grade is primarily attributed to the high risk of bias stemming from the inadequate methodological quality of the studies included. It should be hard to blind participants and personnel during the therapeutic massage process. However, outcome assessor blinding should be considered because it may be useful to control bias to a certain extent. Future studies should pay more attention to research methodology, such as randomization, blinding, estimation of sample size, and so on. Small sample size is also one of the main reasons for the decline of evidence grade. In addition, most studies did not follow up the participants after treatment to observe the rate of recurrence. Only one of the included trials reported adverse events, but none of the included trials reported the recurrent rate or cost-effectiveness. The safety profile of therapeutic massage remains inconclusive, as only one included study reported adverse events. Therefore, future methodologically rigorous, multicenter RCTs with adequately powered sample sizes, standardized treatment protocols and outcome measures, systematic documentation of adverse events, and longitudinal follow-up designs are warranted to comprehensively assess the long-term efficacy, safety, and cost-effectiveness of therapeutic massage integrated with conventional therapies in pediatric FD populations. All included studies in this review were exclusively published in Chinese-language journals, which may introduce potential selection biases and limit the geographic and cultural generalizability of current findings. To enhance the external validity of therapeutic massage efficacy for pediatric functional dyspepsia, future investigations should prioritize multinational RCTs employing rigorous methodologies and standardized protocols. Besides, disseminating these findings through high-impact international journals would strengthen the global applicability of evidence.

### 4.5 Limitations

There are some limitations. First, although all included studies used pediatric therapeutic massage, there will still be some differences in the selection of acupoints for massage in each study. Second, due to limited resources, publications in other languages, such as Japanese or Korean, were not searched. This limitation may have led to the omission of relevant trials published in languages such as Japanese or Korean. Finally, 12 trials in this review were published in Chinese in Chinese journals not included in Medline. It is widely acknowledged that studies published in non-English publications not indexed in Medline may exaggerate the impact of research. Consequently, this review may be influenced by linguistic bias.

## 5 Conclusion

Moderate- to low-certainty evidence suggests that therapeutic massage combined with conventional therapy effectively treats pediatric FD by relieving symptoms and increasing GHRP and leptin levels. Very low certainty of the evidence suggested that therapeutic massage can increase the motolin, gastrin, 5-HT and NPY levels in children with FD. These shows that therapeutic massage combined with conventional therapy may provide symptomatic relief for pediatric FD, but further high-quality trials are needed to confirm its efficacy.

## Data Availability

The original contributions presented in the study are included in the article/supplementary material, further inquiries can be directed to the corresponding author.
